# Belladonna as a Prophylactic

**Published:** 1893-06

**Authors:** C. F. Nichols

**Affiliations:** Boston, Mass.


### BELLADONNA AS A PROPHYLACTIC.

C. F. NICHOLS, M. D., BOSTON, MASS.

The Sister Superior of a large institution here (the home of two hundred and fifty destitute children) sent, six weeks ago for Belladonna, which, she " had heard to be a preventive of scarlatina, thirteen or fourteen children coming down daily."

I sent Swan's DM., to be given in solution every forty-eight hours to sisters and children alike. To-day, January 7th, Sister Mary Louise, Superior, writes that but two children have been touched by any symptoms of illness since beginning the preventive medicine. These two are very slightly disturbed. This seems a test on a large scale.

I may say that in my own practice, I have never known any exposed person to yield to scarlatina, if the interval between doses was as long as twenty-four hours (usually it was forty-eight), and the potency as high as the 200th.

